# The Use of Forceps, &c.

**Published:** 1889-03

**Authors:** 


### THE USE OF THE FORCEPS, THEIR INFLUENCE FOR GOOD AS WELL AS FOR INJURY TO THE PARTURIENT WOMAN.

Dr. Paul F. Mundé. of New York city, in response to special request, sends us his opinion regarding "The use of the forceps, their influence for good as well as for injury to the parturient woman." He says:

"I have always considered the obstetric forceps one of the most useful and beneficial instruments in our armamentarium, one which, in skillful hands, and with proper care and judgment, can *never* do harm either to mother or child, but will often be the only means of saving the life of one or the other, or of both, or of sparing the mother the consequences of delayed delivery and excessive pressure of the soft parts.

"Injury to the mother by the forceps is usually due to their too early application, before the cervix is well dilated, or to the use of excessive or misdirected force in making traction A *cervix may*, but need not, be torn by the forceps. A *perineum* should *never* be injured by the forceps; indeed, the gentle and dexterous guiding of the head over the perineum, so as to permit its gradual distention, is one of the means of preventing that injury. Too much haste in delivering the head with the forceps, is the usual cause of a laceration of the perineum in instrumental deliveries.

"*Abuse of the forceps*, in my opinion, consists in applying them merely to terminate the labor for the sake of the medical attendant; or, when the soft parts are as yet unprepared, or the head is too high, or the pelvis is too narrow.

"The *correct indications* for the use of the forceps, are perfectly clear to a well educated and evenly balanced, conscientious accoucheur. His own convenience certainly should not be an indicationfor their use. But if he knows his skill to be equal to the case, and feels that he can safely deliver the woman with forceps, he should nothesitate, if the conditions being favorable to their use and the labor making no progress, to use them (say two or three hours, without any progress whatever), and thus save the mother unnecessary suffering.

" In short, I think that more harm has been done by too long delay in applying the forceps, and by their unskillful, rude use, than by their too frequent application by skillful hands.

" As for the Tarnier forceps, I confess never to have used them, although I think the principle a correct one, and one which is calculated to save some pioneer in high forceps cases, and ceartainly to save the obstetrician's strength. Their employment is natuıally restricted, more or less, to a high position of the head, chiefly in primiparæ. I have found a Simpson's forceps, with Elliot's screw in the handle to prevent too great compression of the head, perfectly sufficient in my practice, which, obstetrically, is now entirely a consulting one.

" In conclusion, I would say that a slight indentation, or abrasion, of the child's head by the forceps blades cannot always be avoided, particularly in high cases, and such an occurrence should not be considered necessarily as an abuse or unskillful use of the instrument.

"Of course, this subject can be continued indefinitely. I hope I have answered your question with sufficient clearness to suit your purpose."—*Practice.*

